# Identification of a Novel Function for the Chromatin Remodeling Protein ING2 in Muscle Differentiation

**DOI:** 10.1371/journal.pone.0040684

**Published:** 2012-07-12

**Authors:** Shawn A. Eapen, Stuart J. Netherton, Krishna P. Sarker, Lili Deng, Angela Chan, Karl Riabowol, Shirin Bonni

**Affiliations:** Southern Alberta Cancer Research Institute, Departments of Biochemistry and Molecular Biology and Oncology, Faculty of Medicine, University of Calgary, Calgary, Alberta, Canada; McGill University, Canada

## Abstract

The inhibitor of growth (ING) family of zinc-finger plant homeodomain (PHD)-containing chromatin remodeling protein controls gene expression and has been implicated in the regulation of cell proliferation and death. However, the role of ING proteins in cell differentiation remains largely unexplored. Here, we identify an essential function for ING2 in muscle differentiation. We find that knockdown of ING2 by RNA interference (RNAi) blocks the differentiation of C2C12 cells into myotubes, suggesting that ING2 regulates the myogenic differentiation program. We also characterize a mechanism by which ING2 drives muscle differentiation. In structure-function analyses, we find that the leucine zipper motif of ING2 contributes to ING2-dependent muscle differentiation. By contrast, the PHD domain, which recognizes the histone H3K4me3 epigenetic mark, inhibits the ability of ING2 to induce muscle differentiation. We also find that the Sin3A-HDAC1 chromatin remodeling complex, which interacts with ING2, plays a critical role in ING2-dependent muscle differentiation. These findings define a novel function for ING2 in muscle differentiation and bear significant implications for our understanding of the role of the ING protein family in cell differentiation and tumor suppression.

## Introduction

The inhibitor of growth (ING) proteins comprising ING1 to ING5 represents an evolutionary conserved family of chromatin regulators that control gene expression [1], [2], [3], [4]. The expression of ING family members is frequently dysregulated in diverse types of tumors including skin, lung, colorectal and head and neck tumors, suggesting that the ING proteins may play important roles in cancer initiation and progression [3], [5], [6]. These observations also suggest that the ING proteins might play critical roles in cellular homeostasis. However, although members of the ING family have been implicated in the regulation of cell proliferation and apoptosis, with few exceptions [7], the roles of the ING proteins in cell differentiation have remained unknown.

Myogenesis represents an important and established paradigm of cell differentiation in developmental biology [8]. In addition, deregulation of muscle differentiation is thought to underlie pathological conditions including the formation of rhabdomyosarcoma tumors [9]. Therefore, elucidation of the molecular underpinnings of the myogenic differentiation program is critical both for a better understanding of development and disease. The myogenic regulatory factors MyoD and myogenin are members of the basic helix-loop-helix (bHLH) transcription factor family that play key roles in orchestrating myogenesis [10], [11], [12], [13]. Myogenin expression is repressed in undifferentiated myoblasts, and is induced within hours after induction of myogenesis [14]. How chromatin remodeling by transcriptional regulators might control the expression of key myogenesis regulatory factors is of considerable interest.

As critical regulators of chromatin remodeling, the ING proteins are poised to play important roles in cell differentiation. The ING proteins have several conserved regions. Most members of this family have an N-terminal leucine zipper-like motif [4]. The N-terminal region of the ING proteins confers association with transcriptional coregulators including histone deacetylases (HDACs) and histone acetyl transferases (HATs) [15], [16]. The carboxyl terminal region of all ING family members contains a plant homeodomain (PHD), which represents a zinc finger protein-protein interaction domain [17], [18]. Recent studies have shown that the PHD domain binds to histone H3 in a manner dependent on the methylation status of its N-terminal Lysine 4 residue [19], [20], [21]. The ability of the ING proteins to bind transcriptional coregulators and specific histone H3 marks contributes to their ability to regulate gene expression [15].

In this study, we have uncovered a novel function for the ING family protein ING2 in regulation of myogenesis. Knockdown and gain of function analyses reveal that ING2 drives myogenic differentiation. We also identify a mechanism by which ING2 regulates myogenesis. We find that the leucine zipper motif of ING2 contributes to the ability of ING2 to promote muscle differentiation, whereas the PHD domain inhibits ING2-dependent muscle differentiation. Importantly, we find the Sin3A-HDAC1 complex, which interacts with ING2, mediates ING2-dependent muscle differentiation. Collectively, our findings uncover an important role for ING2 in muscle differentiation with significant implications for our understanding of development and tumorigenesis.

## Results

The INGs have emerged in recent years as important regulators of chromatin and gene expression [1]. Although the INGs have been shown to control cell proliferation and apoptosis, their role in cell differentiation has remained largely unknown. Recently, the ING family member ING2 has been implicated in spermatogenesis raising the question whether ING2 regulates differentiation in other systems [7]. We addressed this important question by employing myogenesis as a paradigm for cell differentiation. C2C12 myoblast cells are derived from satellite cells from adult skeletal muscle tissue, and are widely used as a model system in studies of myogenesis as these cells undergo a myogenic genetic program of differentiation similar to primary myoblasts [14], [22]. Under serum-rich growth conditions, C2C12 cells proliferate as undifferentiated mononuclear satellite muscle cells or myoblasts. Incubation in low serum-containing media induces these cells to undergo a temporal differentiation program characterized by cell cycle exit and expression of early myogenic marker and further specialization and fusion of a fraction of these cells to form irreversibly multinucleated myotubes [14], [22]. Muscle differentiation requires G1 arrest and cell cycle exit [14]. Because ING2, can promote cell cycle arrest in diverse cell types [20], [23], [24], we asked whether ING2 might play a role in muscle differentiation.

**Figure 1:**
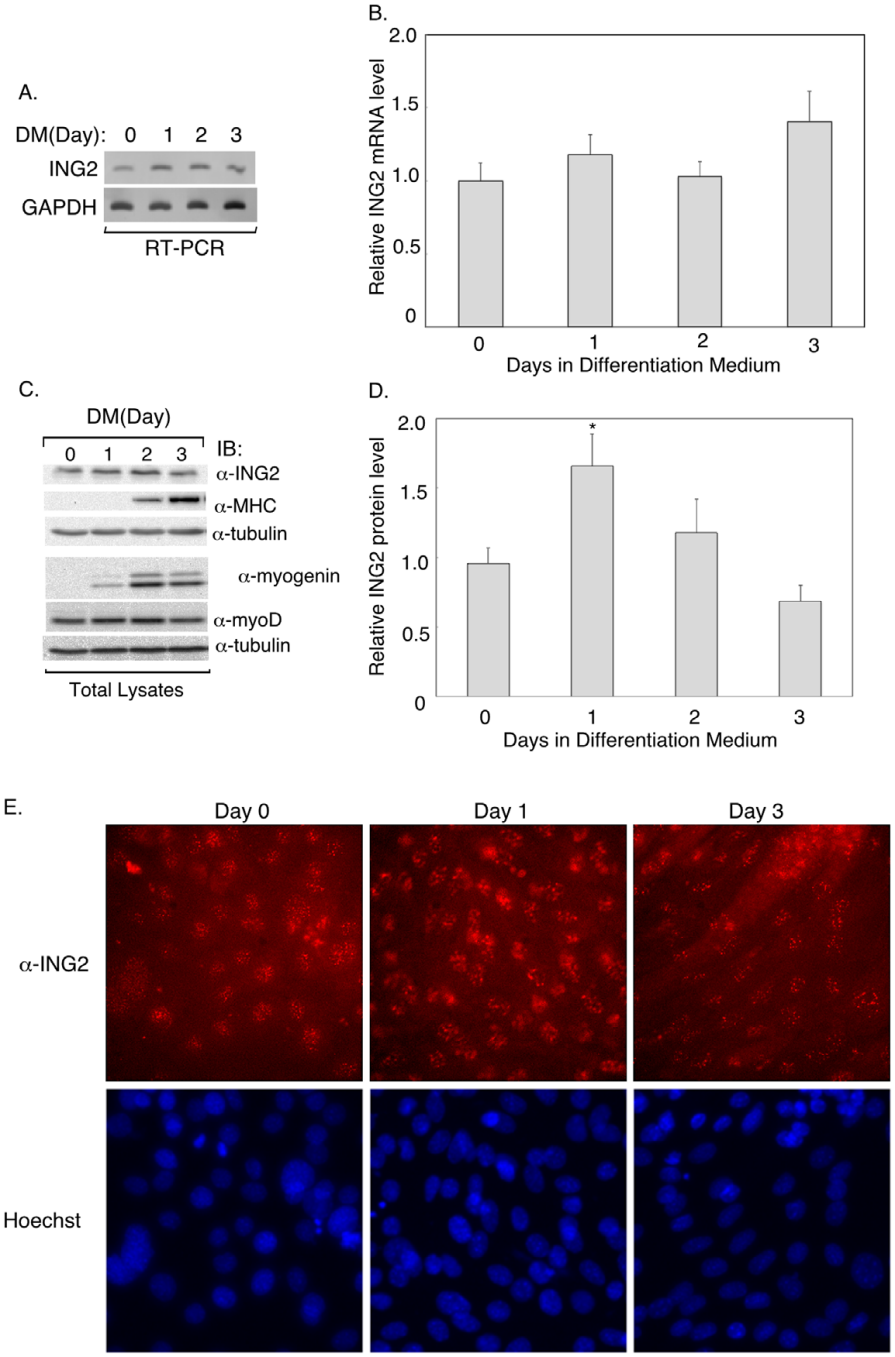
ING2 is expressed in C2C12 myoblast cells under growth and myogenic differentiation conditions. A) Confirmation of identity of quantitative-RT-PCR-amplified ING2 and GAPDH cDNA fragments by end-point RT-PCR. RNA extracts from C2C12 cells incubated for different periods in differentiation media (DM, Day 1, 2, 3) or kept in growth media (DM, Day 0), were reverse transcribed and amplified using specific primers for ING2 and the internal control GAPDH that were also used in the quantitative RT-PCR analysis shown in B (See Materials and Methods). B) RNA extracts of C2C12 cells as described in A were subjected to reverse transcription followed by quantitative real time-PCR to analyze ING2 and GAPDH mRNA levels and determine relative GAPDH-normalized ING2 mRNA level (Materials and Methods). Each column in the bar graph represents the mean (±SEM) of relative GAPDH-normalized ING2 mRNA from seven independent experiments. C) Protein expression profile of ING2 in C2C12 cells. Lysates of C2C12 cells cultured as described in A were subjected to anti-ING2 (11560-AP to detect endogenous ING2 levels) (α-ING2), myosin heavy chain (α-MHC), myogenin (α-myogenin), MyoD (α-MyoD), and tubulin (α-tubulin) immunoblottings (IB), with the latter serving as a loading control. D) ING2 and tubulin levels in immunoblots of lysates of C2C12 myoblasts incubated in growth or differentiation medium as described in A including from ING2 and tubulin immunoblots shown in C were determined using Quantity One Software (Materials and Methods). For each condition, tubulin-normalized ING2 protein level was expressed relative to experimental global average. Each column in the bar graph represents the mean (±SEM) of relative ING2 protein level of five independent experiments. Statistical analyses indicated significant difference in protein levels of ING2 at day 1 as compared to that at day 0 and day 3 of differentiation (P<0.05, ANOVA). E) Subcellular localization of ING2 in C2C12 cells under growth and myogenic differentiation conditions. ING2 localization in cells subcultured in growth media (Day 0) or differentiation media for 1 or 3 days was determined by ING2 indirect immunofluorescence using the anti-ING2 antibody (11560-AP) (α-ING2). Nuclei were visualized using DNA Hoechst staining. Images were taken at X40 magnification. ING2 appears to be localized mainly in the nuclei of single and fused myocytes. Images in A, C and E are from representative experiments that were repeated at least two times.

We first characterized the expression profile of ING2 in undifferentiated and myogenically differentiated C2C12 cells. Quantitative RT-PCR studies showed that ING2 is expressed in cells under growth conditions and upon differentiation (Figure 1A and 1B). Consistent with these results, immunoblotting analyses showed ING2 protein in cells incubated in growth or differentiation media (Figure 1C and 1D). As expected, C2C12 myoblasts expressed the myogenic regulatory factor MyoD, which continued to be expressed in cells under differentiation conditions (Figure 1C). Differentiation induced the expression of the myogenic regulatory factor myogenin (Figure 1C). Myogenin is an early myogenic differentiation marker and is essential for the differentiation of myoblasts into myotubes [25]. We also observed the induction of the terminal myogenic differentiation marker myosin heavy chain (MHC) (Figure 1C). Immunoblotting analyses suggested that ING2 levels may increase modestly during early periods of differentiation and then decrease at later stages (Figures 1C and 1D). Immunofluorescence analyses confirmed that ING2 is expressed in undifferentiated C2C12 cells, as well as in differentiated cells including myotubes (Figure 1E). ING2 displayed mainly punctate nuclear localization in myoblasts and mytubes (Figure 1E). Together, our data show that ING2 is expressed in non-differentiated and myogenically differentiated C2C12 cells.

**Figure 2:**
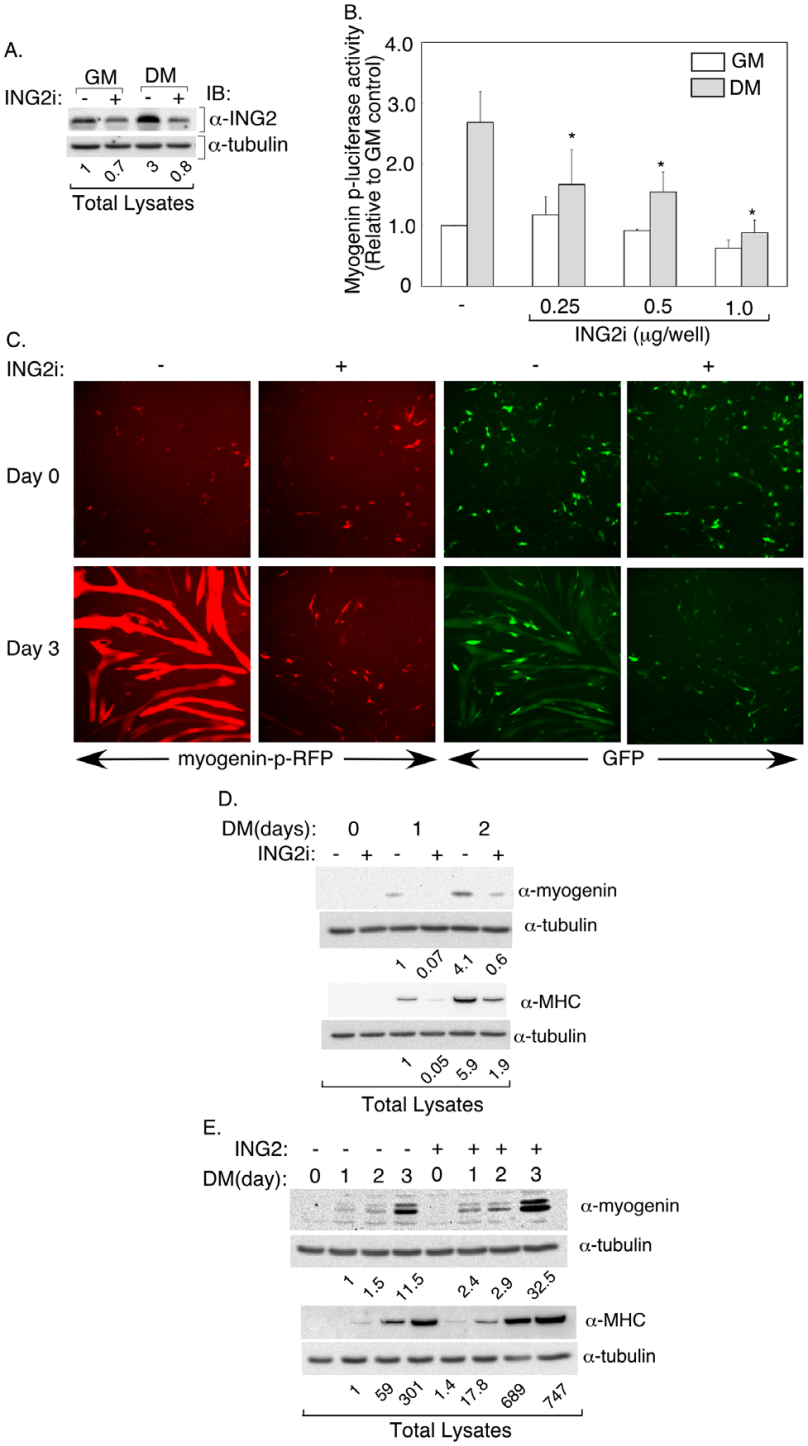
ING2 promotes muscle cell differentiation. A to C) ING2 knockdown negatively regulates myogenesis. A) Knockdown of endogenous ING2 in C2C12 cells. For details refer to Materials and Methods. B) Knockdown of endogenous ING2 represses induction of myogenin-promoter activity during muscle differentiation. Lysates of C2C12 cells transfected with the myogenin-promoter-driven luciferase reporter (myogenin-p-luciferase) vector and the β-galactosidase construct together with a control RNAi (−) or increasing concentrations of ING2 RNAi (ING2i) plasmid, and incubated in growth media (GM) or differentiation media (DM) for 3 days, were subjected to luciferase and β-galactosidase assays as described in Materials and Methods. β-galactosidase-normalized luciferase activity for each transfection was expressed relative to that of control cells cultured in growth media. Each column in the bar graph represents the mean (±SEM) of relative myogenin-p-luciferase activity from three independent experiments. * indicates statistical significant difference (p<0.05, ANOVA) of the ING2 RNAi expressing cells as compared to the control under the same culturing media. C) ING2 RNAi inhibits the ability of C2C12 cells to undergo muscle differentiation. For details see Material and Methods. D) ING2 knockdown induces a reduction in endogenous myogenic differentiation markers. Lysates of C2C12 cells transfected with the control or ING2 RNAi vector and stopped after two days in growth medium (Day 0), or one, or two days after switching to differentiation medium (DM), were subjected to myogenin (α-myogenin), myosin heavy chain (α-MHC), and tubulin (α-tubulin) immunoblottings. Numbers shown below tubulin immunoblots represent tubulin-normalized myogenin levels (upper two panels) or myosin heavy chain levels (lower two panels) expressed relative to respective parameter of the vector control at day 1 of differentiation. No detectable levels of myogenin or myosin heavy chain were apparent in lysates of cells grown under growth condition. E) Stable expression of ING2 promotes muscle differentiation. Lysates of C2C12 cells stably transfected with a control plasmid expressing a resistance marker alone (−) or together with ING2 (+) and kept in growth media (day 0) or incubated in differentiation media (DM) for the indicated time periods were subjected to myogenin (α-myogenin), myosin heavy chain (α-MHC), and tubulin (α-tubulin) immunoblottings, with the latter serving as a loading control. Numbers indicated below are as described in D. Data suggest that ING2 expression produces an overall increase in myogenesis markers. Images in A, C, D, and E are from representative experiments that were repeated at least two times.

The expression of ING2 in C2C12 cells raised the question of whether ING2 might play a role in muscle differentiation. To test this hypothesis, we used RNA interference (RNAi) to characterize ING2 function in myogenesis. We generated a plasmid-based short hairpin (sh) ING2 construct (ING2i) to knockdown mouse ING2 (see Experimental Procedures and [26]). Expression of ING2 shRNAs induced efficient knockdown of ING2 protein in cells including C2C12 myoblasts (Figure S1A and Figure 2A). ING2 knockdown in C2C12 cells persisted during myogenic differentiation (Figure 2A). Because myogenin is a master regulator of muscle differentiation, we determined the effect of ING2 knockdown on the activity of a myogenin promoter-driven luciferase reporter (myogenin-p-luciferase) gene containing a 1.14 kb fragment of the myogenin promoter upstream of the luciferase reporter gene (Experimental Procedures). This promoter fragment includes E-box binding elements for myogenic regulatory factors including MyoD [27]. Incubation of control transfected C2C12 cells under low serum conditions (DM) led to induction of the reporter compared to cells maintained under growth conditions (Figure 2B). However, induction of ING2 knockdown using increasing amounts of the ING2 RNAi plasmid led to a significant reduction in myogenesis-induced luciferase activity, suggesting that endogenous ING2 is important for upregulating myogenin promoter activity during muscle differentiation (Figure 2B). These data also suggested that ING2 might play a role in muscle differentiation.

To test the idea that ING2 plays a role in muscle differentiation, we first established a cell-based assay using a fluorescence microscopy approach. We followed the phenotypes of C2C12 cells transfected with a plasmid containing cDNA encoding tdTomato-red fluorescent protein (RFP) reporter under the control of myogenin promoter elements (myogenin-p-RFP as a marker of myogenic differentiation of the transfected cells). We also included in these transfections a cmv driven-green fluorescent protein (GFP) expression plasmid to serve as a marker for transfection efficiency. Under growth conditions, we found that transfected C2C12 cells expressed GFP, while showing very low myogenin-promoter driven RFP signal reflecting the expected repressed mygenin promoter activity in undifferentiated cells (Figure S1B). Consistent with the undifferentiated phenotypes of these cells, we found that cells lacked specific immunoreactivity for the myosin heavy chain myogenesis marker (Figure S1B). In contrast, cells grown under differentiation conditions showed robust RFP signal which facilitated the visualization of multinucleated myotube formation (Figure S1C). The RFP-labeled myotube represented a subgroup of the myotubes population as they coincided with the myosin heavy chain labeled multinuleated myotubes (Figure S1C). Having established this assay, we then tested the effect of ING2 knockdown on muscle differentiation by cotransfecting C2C12 cells with the myogenin-p-RFP reporter together with the GFP expression plasmid (Figure 2C). GFP expression in cells at Day 0 of differentiation indicated equivalent transfection efficiencies for cells transfected with the ING2 RNAi and control RNAi plasmid (Figure 2C, upper two right panels, and Figure S1D, upper panels). We found that under growth conditions, the control and ING2 knockdown cells behaved similarly with low levels of expression of myogenin-p-RFP whereas GFP was highly expressed in both control and ING2 knockdown cells, reflecting as expected low levels of myogenin promoter-mediated transcription (Figure 2C, upper two left panels versus upper two right panels, and Figure S1D, upper two panels). Upon differentiation, control C2C12 cells displayed robust myotube formation as indicated by multinucleated myogenin-p-RFP (Figure 2C, lower first and third panels, and Figure S1D, lower left panel). Increased intensity of the myogenin-p-RFP fluorescence signal is consistent with the expected enhanced myogenin promoter-mediated transcription during myogenic conversion (Figure 2C and Figures S1C and S1D). By contrast, ING2 knockdown drastically reduced the level of RFP expression as well as myotube formation (Figure 2C and Figure S1D). Indirect immunofluorescence to visualize the terminal muscle differentiation marker myosin heavy chain also demonstrated that ING2 knockdown led to a consistent reduction in the number and size of myotubes (Figure S1E). Consistent with the indirect immunofluorescence data, we found in immunoblotting studies ING2 knockdown led to reduction in protein levels of myogenin and myosin heavy chain (Figure 2D). In complementary analyses, we found that stable expression of ING2 in C2C12 cells enhanced muscle differentiation as indicated by increased levels of myogenin and myosin heavy chain expression (Figure 2E). Collectively, our findings suggest that ING2 promotes muscle differentiation.

**Figure 3:**
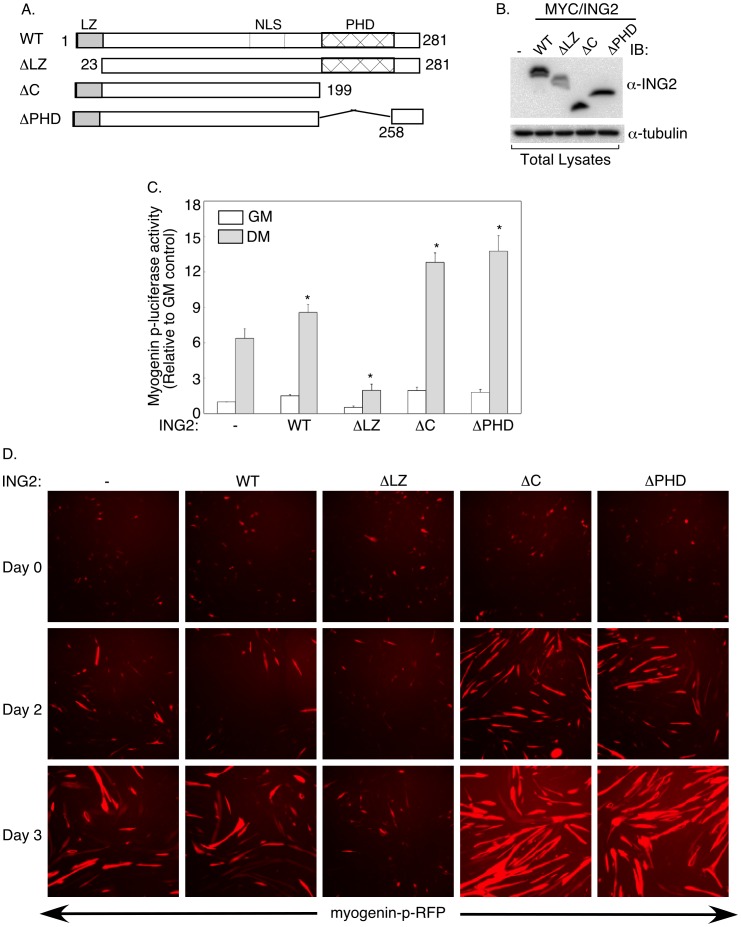
The leucine zipper motif (LZ) and plant homeodomain (PHD) domain define important molecular determinants in ING2’s ability to promote muscle differentiation. A) A schematic representation of the wild type protein ING2 (WT) and each of ING2 ΔLZ, ΔC, and ΔPHD, that lack, respectively, amino acid residues 1–23 (leucine zipper motif (LZ), gray shaded rectangle), 199–281 (PHD-containing carboxy terminal region, cross-hatched and clear rectangles) and 199–258 (PHD, cross-hatched rectangle). The relative location of the nuclear localization signal (NLS) is also indicated. B) Lysates of C2C12 myoblast cells transfected with expression plasmids containing cDNA encoding ING2 (MYC/ING2) WT or the deletion mutant ΔLZ, ΔC or ΔPHD, as described in A, were immunobloted with anti-ING2 (16186-1-AP) (α-ING2) and anti-tubulin (α-tubulin) antibodies (IB), with the latter serving as a loading control. The anti-ING2 (16186-1-AP) antibody is efficient in detecting overexpressed wild type and mutant ING2 but not endogenous ING2. C) Lysates of cells transfected with the myogenin-p-luciferase reporter construct and the β-galactosidase encoding vector together with an empty expression vector (−) or one encoding wild type ING2 (WT), or the ING2 deletion mutant ΔLZ, ΔC, or ΔPHD, and incubated with growth media (GM) or differentiation media (DM) for 3 days, were subjected to luciferase and β-galactosidase assays and analyses as described in Figure 2B. Each column in the bar graph represents the mean (± SEM) of relative myogenin-p-luciferase activity from five independent experiments. * indicates statistical significant difference (p<0.05, ANOVA) of the ING2 expressing cells incubated with differentiation medium versus that of the control transfectant grown in the same conditions. D) Cells were transfected with the myogenin-p-RFP reporter gene construct, to follow myogenic differentiation of transfected cells, and GFP encoding pEGFP (N1) plasmid, to detect transfected cells, together with an empty expression vector (−) or one encoding wild type ING2 (WT), or ING2 variant ΔLZ, ΔC, or ΔPHD. Two days post transfection, cells were either fixed (Day 0), or incubated with differentiation media for two days or three days prior to fixing, labeled with the Hoechst stain, and subjected to fluorescence microscopy to visualize myogenin-p-RFP, GFP (Figure S2B, green) and nuclei (Figure S2B, blue). A representative field for the myogenein-p-RFP (red) signal for each condition is shown from an experiment that was repeated five independent times.

Next, we characterized the mechanism by which ING2 regulates muscle differentiation. We first focused on mapping the regions within ING2 that regulate this biological response. We compared the myogenic effect of wild type ING2 (WT) to that of the deletion mutant ING2 (ΔLZ) lacking amino acids 1 to 23 encompassing a lecuine zipper motif, ING2 (ΔC) missing carboxyl-terminal amino acid residues 199 to 281 containing the PHD domain, or ING2 (ΔPHD) lacking amino acid residues 199 to 258 corresponding to the PHD domain (Figure 3A). Immunoblotting of transfected C2C12 cell lysates confirmed expression of wild type ING2 and each of the three deletion mutants of ING2 lacking the leucine zipper motif, the PHD-domain-containing carboxyl-terminal region, or only the PHD domain (Figure 3B). Indirect immunofluorescence showed similar predominant nuclear localization of the wild type ING2 and the three ING2 deletion mutants, consistent with all variants retaining their nuclear localization sequences (Figure S2A) [1], [28]. Expression of wild type ING2 led to a modest but significant increase in myogenin-mediated transcription in differentiating C2C12 cells (Figure 3C). Strikingly, we found that expression of ING2 (ΔZ), lacking the leucine zipper motif, blocked myogenin promoter-mediated transcription in differentiating C2C12 cells, suggesting that ING2 (ΔLZ) acts in a dominant negative manner to block myogenin expression. In contrast, deletion of the PHD domain, or the C-terminal region of ING2 increased the ability of ING2 to enhance myogenin promoter activity (Figure 3C). Consistent with these data, we found that ING2 (ΔLZ) profoundly inhibited, whereas ING2 (ΔC) or (ΔPHD) increased myotube formation and myogenin-p-RFP intensity (Figure 3D, and Figures S2C and S2D). Qualitative analysis of nuclear and GFP fluorescence profiles of ING2 (ΔLZ) expressing cells revealed no observable differences as compared to control or wild type- or other deletion mutant ING2-expressing cells, suggesting that the ability of ING2 (ΔLZ) to inhibit muscle differentiation is not due to apoptosis (Figure S2B). Altogether, these data suggest that the leucine zipper motif contributes to ING2 function in muscle differentiation, whereas the PHD domain inhibits the ability of ING2 to promote myogenesis.

**Figure 4:**
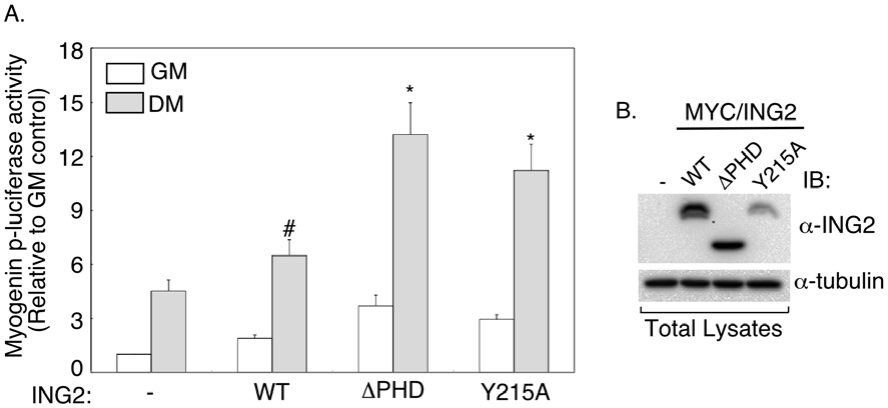
ING2-Lysine-4-di/trimethylated histone H3 binding region inhibits ING2-dependent muscle differentiation. A) C2C12 cells transfected with the myogenin-p-driven luciferase reporter gene construct and the β-galactosidase plasmid together with an empty expression vector (−) or one encoding wild type ING2 (WT), a ΔPHD mutant ING2, or a Tyrosine 215 to alanine mutant ING2 protein (Y215A), were left in growth medium (GM) or incubated for three days in differentiation medium (DM) and subjected to luciferase and β-galactosidase assays and analysis as described in Figure 2B. The values shown in the bar graph are the mean (±SEM) of seven independent experiments. * indicates significant difference as compared to differentiation condition control (p<0.05, ANOVA). # indicates significant difference as compared to the differentiation control (p<0.05, two-tailed, paired t-test) B) The expression levels of the wild type and each of the ΔPHD and Y215A mutant ING2 were confirmed by immunoblotting with anti-ING2 (16186-1-AP) (α-ING2) and anti-tubulin (α-tubulin) antibodies.

The ING2 PHD domain interacts with histone H3 in a Lysine 4 methylation-sensitive manner [20], [21]. Tyrosine 215 within the PHD domain is critical for ING2’s interaction with histone H3 that is di- or tri-methylated at Lysine 4 [20]. We tested the effect of mutation of ING2 in which Tyrosine 215 was replaced with alanine (Y215A) on the ability of ING2 to induce myogenin promoter-mediated transcription (Figure 4A and [20]). In control immunoblotting and indirect immunofluorescence analyses, we confirmed that the Y215A mutant ING2 protein was expressed and localized to the nucleus in cells (Figure 4B and Figure S2A). Importantly, we found that just as with deletion of the PHD domain, the Y125A mutation increased the ability of ING2 to enhance myogenin promoter activity during muscle differentiation (Figure 4A). Together, these results suggest that the interaction of ING2 with trimethylated histone H3 inhibits the ability of ING2 to promote muscle differentiation.

**Figure 5:**
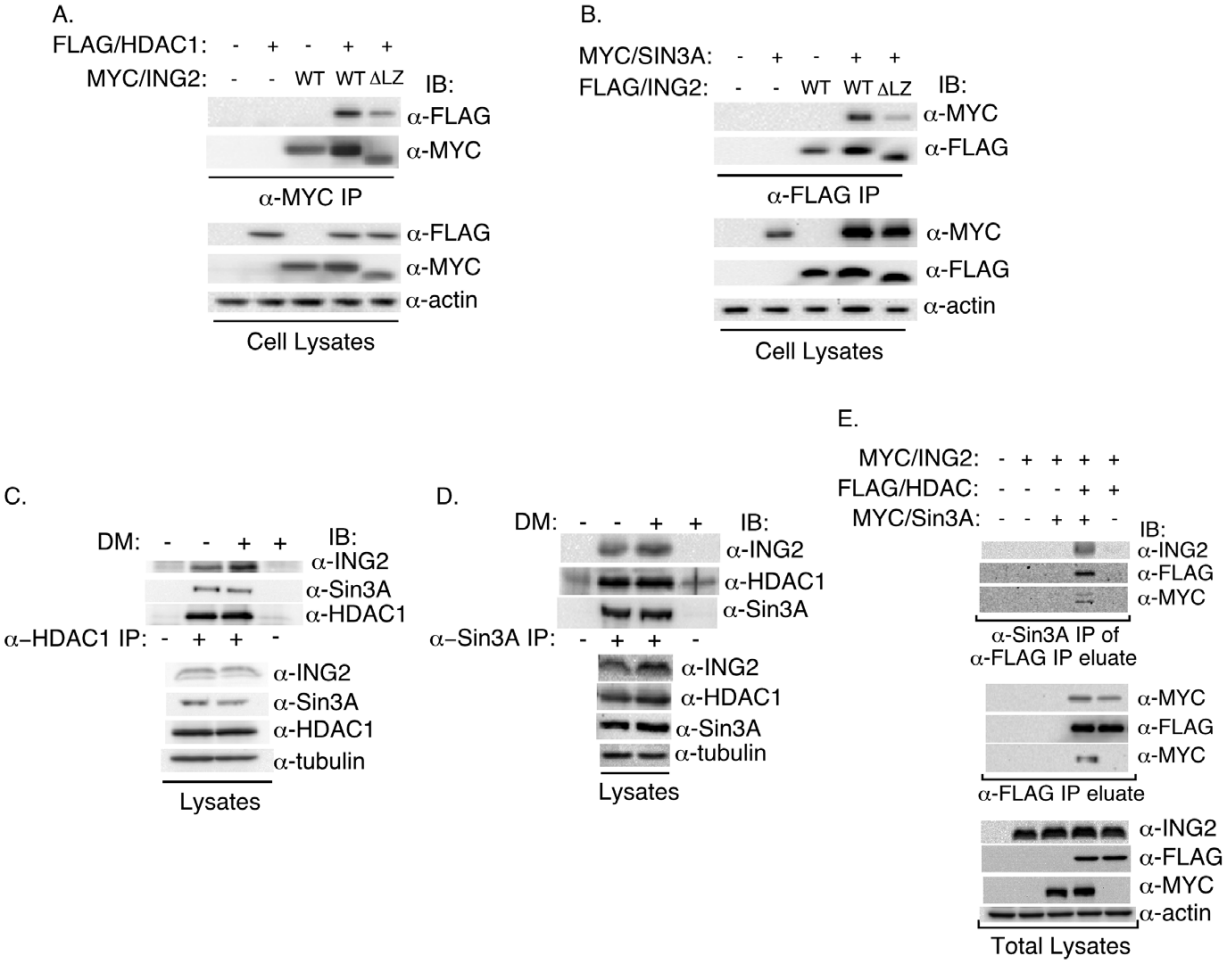
ING2 associates via its leucine zipper motif with HDAC1 and Sin3A. ING2 associates with HDAC1 (A) and Sin3A (B) and this interaction is reduced by deletion of ING2 amino-terminal leucine zipper motif. A) Lysates of 293T cells cotransfected with different combinations of HDAC1 (FLAG/HDAC1), and ING2 (MYC/ING2) wild type (WT) or leucine zipper motif deleted (ΔLZ) form, were subjected to ING2 immunoprecipitation (α-MYC IP) followed by sequential HDAC1 (α-FLAG) and ING2 (α-MYC) immunoblottings (IB). Total lysates were immunblotted for HDAC1 (α-FLAG), ING2 (α-MYC), and actin (α-actin), with the latter serving as loading control. B) 293T cells cotransfected with Sin3A (MYC/Sin3A), and ING2 (FLAG/ING2), wild type (WT) or deleted in leucine zipper motif (ΔLZ), were lysed, and subjected to ING2 (α-FLAG) immunoprecipitation followed by Sin3A (α-MYC) and ING2 (α-FLAG) immunoblottings. Lysates were assessed by immunoblottings for expression of Sin3A (α-MYC), ING2 (α-FLAG) and the loading control actin (α-actin). ING2 associates with HDAC1 (C) and Sin3A (D) in C2C12 cells under growth and myogenic-differentiation conditions. C) Lysates from C2C12 cells incubated in differentiation media (DM, +) for one day or in growth media (DM, −), were subjected to HDAC1 immunoprecipitation (α-HDAC1 IP, +) or an irrelevant antibody (α-HDAC1 IP, −) followed by immunoblotting with ING2 (11560-AP) (α-ING2), Sin3A (α-Sin3A), and HDAC1 (α-HDAC1) antibodies. Total lysates were subjected to ING2, Sin3A, HDAC1, and tubulin immunoblottings, with the latter serving as a loading control. D) Lysates from C2C12 cells as in C were subjected to Sin3A immunoprecipitation (α-Sin3A IP, +) or an irrelevant antibody (α-Sin3A IP, −) followed by immunoblotting (IB) with anti-ING2 (11560-AP) (α-ING2), HDAC1 (α-HDAC1), and Sin3A (α-Sin3A) antibodies. Total lysates were assessed for ING2, HDAC1, Sin3A, and tubulin expression as in C. E) ING2 forms a complex with HDAC1 and Sin3A. Lysates of 293T cells transfected with an empty expression vector (−), or one encoding MYC-tagged ING2 alone, or together with MYC-tagged Sin3A, FLAG-tagged HDAC1, alone or together were subjected to sequential anti-FLAG immunoprecipitation, FLAG peptide elution, and Sin3A immunoprecipitation of FLAG-eluate immunocomplexes (α-Sin3A IP of α-FLAG IP eluate), followed by ING2 (α-ING2), HDAC1 (α-FLAG) and Sin3A (α-MYC) immunoblotting. ING2 and Sin3A coimmunoprecipitation by HDAC1 were confirmed by subjecting a fraction of FLAG IP eluate to ING2 (α-MYC, upper panel) and Sin3A (α-MYC, lower panel) immunoblotting. Lysates were immunoblotted for assessing levels of ING2, HDAC1, Sin3A, and the loading control actin as shown. Anti-ING2 (11560-AP) antibody was used to detect ING2 in the HDAC1-Sin3A immunocomplexes and lysates. Low exposure was used for scans of ING2 in total lysates to avoid endogenous ING2 detection. Scan shown in A to E are from representative experiments that were repeated at least two times.

Next, we characterized the potential mechanism by which the leucine zipper motif might regulate ING2 function in muscle differentiation. ING2 operates in concert with the Sin3A-HDAC1/2 histone modifying complex in the regulation of gene expression [15], [29]. We confirmed that exogenous ING2 interacts with co-expressed HDAC1 and Sin3A in cells (Figures 5A and 5B). We also found that endogenous ING2 may form a complex with endogenous HDAC1 and Sin3A in C2C12 myoblasts (Figures 5C and 5D). Sequential coimmunoprecipitation analyses suggested that ING2 can exist as a multiprotein complex together with HDAC1 and Sin3A (Figure 5E and Figures S3C and S3D). Interestingly, in structure-function analyses, we found that deletion of the leucine zipper motif reduced the ability of ING2 to interact with HDAC1 and Sin3A (Figures 5A, 5B and Figures S3A and S3B). These results suggest that the leucine zipper motif, which is critical for ING2 function in myogenesis, endows ING2 with the ability to interact with the Sin3A-HDAC1 complex.

**Figure 6:**
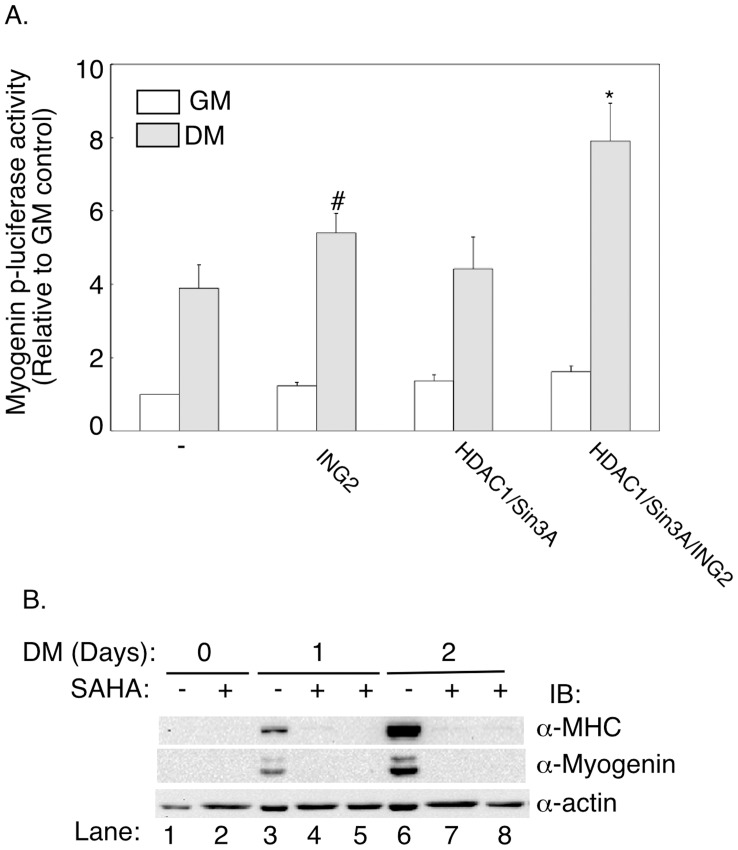
The HDAC1/Sin3A complex promotes muscle cell differentiation. A) Coexpression of Sin3A and HDAC1 potentiates ING2’s promotion of myogenin-promoter activity during myogenesis. C2C12 cells cotransfected with myogenin-promoter-driven luciferase reporter (myogenin-p-luciferase) and β-galactosidase reporter constructs, together with an empty vector (−), ING2, low amounts Sin3A and HDAC1 expressing vectors, alone or together, were kept under growth (GM) or differentiation conditions for three days (DM). Cells were lysed and subjected to luciferase and β-galactosidase assays and analysis as described in Figure 2B. Each column in the bar graph represents the mean (±SEM) of relative luciferase activity from six independent experiments. * indicates significant difference from the control, ING2, and Sin3A and HDAC1 groups grown under differentiation (P<0.05, ANOVA). # indicates significant difference from the differentiation control (P<0.05, two-tailed unpaired t-test). B) The HDAC inhibitor SAHA blocks myogenic differentiation. Lysates of C2C12 cells incubated in growth media (Lanes 1, and 2) or differentiation media (Lanes 3 to 8) in the absence (Lanes 1, 3, and 6), or presence (Lanes 2,4,5,7, and 8) of SAHA (3.75 µM), were immunoblotted (IB) for myosin heavy chain (α-MHC), myogenin (α-myogenin), and actin (α-actin), as a loading control. Lysates analyzed in Lanes 4 and 7 are from cells to which SAHA was added one day prior to incubation in differentiation media, and lysates of Lanes 5 and 8 are from cells that were incubated simultaneously with SAHA and differentiation media. Under all conditions, SAHA inhibits the ability of differentiation stimuli to promote myogenesis as indicated by myogenin and myosin heavy chain expression.

The finding that ING2 interacts via its leucine zipper motif with Sin3A and HDAC1 led us to investigate the role of Sin3A and HDAC1 in ING2-dependent muscle differentiation. In co-expression studies in C2C12 myoblasts, we found that expression of exogenous ING2 together with Sin3A and HDAC1 synergistically increased myogenenin promoter-mediated transcription (Figure 6A). In complementary experiments, we tested the effect of the HDAC inhibitor SAHA on muscle differentiation. Incubation of C2C12 myoblasts with SAHA effectively inhibited the ability of the cells to differentiate into muscle cells reflected by dramatic reduction in myogenin and myosin heavy chain levels (Figure 6B). Inhibition of these muscle proteins was evident whether SAHA was added one day prior to or at the start of differentiation. These results support the conclusion that the Sin3A-HDAC1 chromatin remodeling complex promotes myogenesis. Collectively, our findings suggest that ING2 plays a critical role in muscle differentiation in a manner dependent on its leucine zipper motif and the Sin3A-HDAC1 complex.

## Discussion

In this study, we have discovered a novel function for the chromatin remodeling protein ING2 in cell differentiation. Results of loss and gain of function studies suggest that ING2 promotes the differentiation of C2C12 myoblast into muscle cells. We have also characterized the molecular basis of ING2-dependent muscle differentiation. Structure-function analyses reveal that the leucine zipper motif of ING2 contributes to the ability of ING2 to promote muscle differentiation. In contrast, the PHD domain, which recognizes the H3K4me3 chromatin mark, inhibits ING2-dependent muscle differentiation. Finally, we have found that the Sin3A-HDAC1 chromatin-remodeling complex interacts with ING2 via its leucine zipper motif and thereby promotes muscle differentiation. Taken together, our findings define ING2 and the Sin3A-HDAC1 chromatin remodeling complexes as a novel epigenetic mechanism that promotes muscle differentiation, with important implications for our understanding of ING functions in cell differentiation and tumor suppression.

ING2 controls diverse cellular responses including cell proliferation and survival [16], [20], [23], [24]. ING2 promotes cell cycle arrest or apoptosis in fibroblasts and epithelial cells upon exposure of distinct stimuli including genotoxic stress signals and growth factors [16], [20], [23], [24]. The ING2 related proteins ING1 and ING4 are reported to regulate cell cycle progression, cell death and replicative senescence [1], [2], [3], [4]. Our finding that ING2 promotes muscle differentiation adds a new dimension to ING functions in cellular response. In future studies, it will be interesting to determine whether ING2 function in myogenesis is regulated by extrinsic cues. ING2 function in muscle cell differentiation suggests that ING2 may play an important role in tissue development. Corroborating our findings is the reported role of ING2 in meiosis and spermatocyte differentiation [7]. The finding that ING2 promotes cells differentiation may also explain at a cellular level how ING2 operates in a tumor suppressive manner. Future studies should also examine the role of other ING members on muscle differentiation. ING2 knockdown in C2C12 cells dramatically inhibited myotube formation, suggesting little if any compensatory effects by other ING family members in these cells. However, ING2 may operate together with other ING family members in muscle differentiation in vivo. In particular, it is tempting to speculate that ING2 and the closely related family member ING1 may operate together to promote muscle differentiation in vivo.

The cyclin dependent kinase inhibitor p21 has been reported to be regulated by ING2 [23]. p21 is induced during muscle differentiation and is critical for cell cycle arrest [14]. Thus, it is possible p21 may contribute to the ability of ING2 to control muscle differentiation. Muscle differentiating C2C12 myoblasts appear to show initial increase in myogenin expression followed by p21 induction [14]. This temporal pattern of gene expression is consistent with the possibility that ING2 may promote muscle differentiation via p21-independent mechanisms.

The finding that the leucine zipper motif contributes to ING2-dependent-muscle differentiation bears important implications for our understanding of the mechanism of ING2 function. Intriguingly, the leucine-zipper-motif containing region of ING2 has been reported to be important for ING2 in nuclear excision DNA repair and apoptosis [16], while it may not contribute to ING2’s regulation of cell proliferation [21], [24]. These observations raise interesting parallels between ING2 function in the control of DNA repair, apoptosis, and cell differentiation.

We have also found that ING2 interacts via its leucine zipper motif with the Sin3A-HDAC1 complex and thereby promotes muscle differentiation. These findings suggest a novel role for Sin3A-HDAC1 in myogenesis. In future studies, it will be important to determine how the Sin3A-HDAC1 complex promotes muscle differentiation. Interestingly, a subset of the ING2-Sin3A complexes can exist as part the BRG1-based SWI/SNF chromatin remodeling complex [15], raising the possibility that ATP-dependent relaxation of chromatin by this multi-protein complex may facilitate the entry of transcription factors, cofactors, and RNA polymerase II to promoters of myogenic genes.

In conclusion, our study defines a novel role for ING2 in muscle differentiation and provides a potential mechanism by which ING2 affects myogenesis. These findings have important implications for ING function in cell differentiation and tumor suppression.

## Materials and Methods

### Ethics Statement

N/A.

### Plasmids

pCMV5 plasmid containing cDNA encoding MYC or FLAG epitope tagged-wild type human ING2 was generated by a PCR-approach using pCI-ING2 as a template. Expression constructs of deletion mutants of ING2 cDNA were generated by PCR and subcloning the amplified products into the pCMV5 vector using convenient restriction endonuclease sites. To generate a CMV-based expression construct encoding ING2 in which Tyrosine 215 was converted to alanine, ING2 cDNA was subjected to site-directed mutagenesis via a nested PCR method. The pU6 control vector containing the promoter of the mouse U6 non-coding small nuclear RNA (snRNA) was used to generate the ING2 RNAi vector [26]. The ING2 RNAi vector expressing a short hairpin RNA under the control of the U6 promoter was constructed using the forward primer (GCCAAGAATTGGGAGATGAAA**CAAGTTAAC**TTTCATCTCCCAATTCTTGGTT TTTG) and the reverse primer (AATTCAAAAACCAAGAATTGGGAGATGAAA**GTTAACT TG**TTTCATCTCCCAATTCTTGGC) as described in detail elsewhere [26]. The ING2 RNAi plasmid was designed to target the sequence CCAAGAATTGGGAGATGAAA corresponding to nucleotides 281 to 301 of the mouse ING2 mRNA. The myogenin-promoter driven-luciferase reporter (myogenin-p-luciferase) construct [27] was used to generate the myogenin-promoter-tdTomato red fluorescent protein (myogenin-p-RFP) by replacing the luciferase reporter with the tdTomato cDNA [30] **.** Identities of the plasmids were confirmed by restriction site digestion, DNA sequencing and western blotting.

### Cell Cultures and Transfections

Mouse C2C12 myoblasts were grown in Dulbecco’s modified essential medium (DMEM) with high glucose, L-glutamine and sodium pyruvate, supplemented with 10% fetal bovine serum (FBS). Human 293T kidney epithelial cells (American Type Culture Collection (ATCC)) and C2C12 cells (ATCC) were generous gifts from Dr. Jeffrey Wrana. 293T cells were grown in 10% FBS containing-high glucose and L-glutamine-DMEM. 293T cells were transfected using the calcium chloride method. C2C12 cells were transfected using a liposome-based FuGene® 6 Transfection Reagent (Roche Applied Biosciences) or TransIT LT1 reagent (Mirus Bio Corporation) according to the manufacturer’s instructions. C2C12 cells were induced to differentiate into the myogenic lineage by feeding the cells with differentiation media (DM) consisting of DMEM supplemented with 2% horse serum [31]. For experiments examining the effect of HDAC blockade on muscle differentiation, cells were incubated with the HDAC1/2 inhibitor suberoylanilide hydroxamic acid (Vorinostat (SAHA), Selleck) at a final concentration of 3.75 µM, or an equivalent volume of the vehicle dimethyl sulfoxide (DMSO, SIGMA) as described in Figure 6B legend.

### Cell Extract Preparation and Immunoblotting

To obtain cellular extracts for western blot analyses, cells were incubated for 20 minutes at 4°C in TNTE lysis buffer (50 mM Tris, 150 mM NaCl, 1 mM EDTA, 0.5% [v/v] Triton-X-100) containing protease and phosphatase inhibitors. Lysates were centrifuged at 15,000×g for 10 minutes at 4°C, and small aliquots were subjected to protein concentration determination using Bradford-based protein assays (Bio-Rad Laboratories). Protein mixtures in the cell lysates were resolved by SDS-PAGE and were transferred electrophoretically onto nitrocellulose membranes (Bio-Rad Laboratories). After blocking, the blots were incubated with mouse anti-myc (9E10, Cedarlane), rabbit anti-ING2 (11560-1-AP) and (16186-1-AP) antibodies (Proteintech), mouse anti-myogenin (Developmental Studies Hybridoma Bank (DSHB)), mouse anti-myosin heavy chain (MHC) (DSHB), rabbit anti-MyoD (Santa Cruz), rabbit anti-actin (Sigma), or mouse anti-tubulin (Santa Cruz), as the primary antibody and HRP-conjugated donkey anti-mouse or anti-rabbit IgG as secondary (Amersham) followed by ECL™ and signal detection using a VersaDoc 5000 Imager (Bio-Rad Laboratories). Densitometry was performed using Quantity One® software (Bio-Rad Laboratories). We found that anti-ING2 antibody (11560-1-AP) to be more sensitive and efficient than anti-ING2 antibody (161861-AP) in detecting relatively low levels of ING2. We routinely used the antibody (11560-AP) for visualizing endogenous ING2 protein levels, while we used the antibody (161861-AP) for detection of exogenous ING2 in C2C12 cells.

In experiments investigating the effect of ING2 RNAi on endogenous ING2 (Figure 2A), lysates of C2C12 cells two days following transfection with the RNAi control vector (ING2i, −) or the ING2 RNAi vector (ING2i, +), and culturing in growth medium (GM), or switching to differentiation medium for a day (DM), were subjected to immunoblotting with the anti-ING2 (11560-AP) (α-ING2), and anti-tubulin (α-tubulin) antibodies. ING2 and tubulin levels were quantified as described above and in Figure 1D. Each value below each lane of Figure 2A represents tubulin-normalized ING2 expressed relative to the vector control cells grown under growth medium. Considering transfection efficiencies of approximately 20 to 30%, ING2 RNAi induces efficient knockdown of endogenous ING2 in transfected cells. ING2 knockdown persisted for several days in differentiation.

### Luciferase Reporter Assay

C2C12 cells seeded in 24-well plates at a density of 8×10^4^ cells per mL were transfected the following day with the myogenin-p-luciferase reporter, and a CMV-based β-galactosidase constructs [31], together with additional vectors as indicated in the legends of Figures 2B, 3C, and 4A. Forty hours post-transfection, cells were either kept in growth medium (GM) or switched to differentiation medium (DM) for 3 days [31]. Cells were lysed in Reporter Lysis Buffer (Promega) and subjected to luciferase and β-galactosidase assays, and luciferase activity (Relative Light Units, RLU) was normalized to β-galactosidase activity to control for variation in transfection efficiency [26], [31]. Each experimental condition was carried out in triplicate. Experiments were repeated independently at least three times.

### Quantitative Reverse Transcriptase-PCR

RNA from C2C12 cells kept in GM or shifted to DM was extracted using Trizol lysis reagent (TRIzol-Gibco) following the manufacturer’s protocol. DNase-treated RNA was reverse transcribed to generate Poly (A)-cDNA using reverse transcriptase SuperScript II (Invitrogen Life Technologies) and oligo-(dT)12–18 (Amersham Biosciences). ING2 and Glyceraldehyde 3-phosphate dehydrogenase (GAPDH) cDNA fragments were subjected to quantitative real time-PCR using the polyA-cDNA as template and gene-specific ING2 (forward- TGAAAGTGAGCG AGCCTCAGACAA and reverse- TCGGTGGTTGATCATCACAGTCGT) and GAPDH (forward- TCAACAGCAACTCCCACTCTTCCA and reverse-ACCCTGTTGCTGTAGCCGT ATTCA) primers (Rotor-Gene: Corbett Research). Cycle threshold (Ct) of ING2 RT-PCR to GAPDH's Ct ratio (delta (Δ) Ct) was used in the formula (2^ Λ(−ΔCt)^) to obtain relative ING2 mRNA levels. For each experiment, the relative ING2 mRNA level in each sample was normalized to global average. PCR products of 149 and 115 base-pairs (bp) were generated for ING2 and GAPDH, respectively, which were confirmed by end point RT-PCR (Figure 1A). The amplified fragments of end point RT-PCR were resolved using acrylamide gel electropheresis, stained with 1 mg/ml ethidium bromide, and scanned using the VersaDoc 5000 Imager.

### Indirect Immunofluorescence/Fluorescence and RFP Myotube Assay

C2C12 cells seeded in 24 well plates at a concentration of 6×10^4^ cells per mL, were transfected with the constructs indicated in the figure legends. After different times in DM, cells were fixed (4% formaldehyde), permeabilized (0.2% Triton X-100), and incubated with Hoechst 33342 DNA fluorescent dye (Invitrogen) alone, or together with specific primary and fluorescent-tagged secondary antibodies (see figure legends). Cells were imaged using the KineticScan-HCS-Reader that is equipped with a Carl Zeiss Axiom × microscope and visualized with KineticScan-Reader software (Cellomics, Inc, Pittsburgh, PA, [32]). In myogenin-RFP-based transfection experiments, myotubes were identified by the KineticScan-Reader based on RFP expression (Cell Morphology-Myotube BioApplication). Within each identified myotube, the number of nuclei and/or the intensity of the myogenin-p-RFP fluorescence were assessed. Each experimental condition was done in triplicate, and experiments were repeated at least three independent times, analyzing minimum of 500 myotubes per well per experiment to properly deal with any potential intra- and inter-experimental variability in the myotube formation assay. Control cells under growth and differentiation conditions were included in each experiment to serve as appropriate references for comparing other experimental groups. This approach helped overcome potential variability in the degree of myotube formation in control cells, for example as in Figures 2C and 3D.

For experiments investigating the effect of ING2 RNAi on muscle differentiation (Figure 2C), cells transfected with the myogenin-promoter driven tdTomato red fluorescent protein reporter (myogenin-p-RFP) vector, and the enhanced green fluorescent protein pEGFP (N1) construct (Clontech) (GFP), as indicators of muscle differentiation and transfection efficiency, respectively, together with a control (−) or ING2 RNAi (+) plasmid were left in growth media for two days post transfection and were either fixed then (Day 0) or three days after incubating in differentiation medium (Day 3). Nuclei of fixed cells were stained with Hoechst dye, and cells were scanned for myogenein-p-RFP (red), GFP (green) and Hoechst (blue, Figure S1D) signals using fluorescence microscopy. All images were captured at a constant time of exposure to allow direct comparison of RFP intensity and help ascertain the effect of the ING2 knockdown on induction of myogenin promoter activity and hence on muscle differentiation.

### Statistical Analysis

Independent experiments’ mean values were analyzed by student-t-test or analysis of variance (ANOVA) followed by post hoc tests to determine statistical significance (p<0.05).

## Supporting Information

Figure S1 **ING2 knockdown negatively regulates myogenesis.** A) ING2 RNAi plasmid induces ING2 knockdown. Lysates of 293T cells transfected with empty vector (FLAG/mING2, −) or mouse ING2 expressing vector (FLAG/mING2, +) together with the RNAi vector control (ING2i, −) or ING2 RNAi (ING2i, +) plasmid, were subjected to FLAG and actin immunoblottings, with the latter serving as loading control. ING2 RNAi induces efficient reduction in ING2 protein levels. B and C) The myogenin-p-RFP reporter as a marker of myogenic differentiation in transfected cells. C2C12 cells transfected with a vector containing cDNA encoding the tdtomato-red fluorescent protein (RFP) under the control of the myogenin promoter together with the pEGFP(N1) plasmid to express green fluorescent protein (GFP) under the control of CMV promoter, were left in growth medium for another two days, and fixed then (B), or three days after switching to myogenic differentiation medium (C). Cells were labeled for the terminal differentiation marker myosin heavy chain (MHC) using indirect immunofluorescence and for nuclei using Hoechst DNA stain, and visualized by fluorescence microscopy (Material and Methods). B) Under growth condition only a small fraction of transfected cells (GFP) express RFP due to low myogenic differentiation as indicated by low myosin heavy chain signal. C) Upon differentiation, RFP expression is enhanced drastically and appears to be enriched in multinucleated myotubes as reflected by overlap with a subpopulation of the myosin heavy chain labelled myotubes. D) Scans relating to RFP and GFP fluorescence images of cells transfected with control and ING2 RNAi shown in Figure 2C. The scans in upper panel show cell numbers under growth conditions (Day 0) as indicated by Hoechst staining. The lower two panels are merged images of myogenin-p-RFP (same images as in lower panels of Figure 2C) and nuclei fluorescent signals of cells transfected with vector control RNAi (left panel) or with ING2 RNAi plasmid (right panel). RFP expression reflecting multinucleated and hence fused myotubes derived from the control RNAi transfected cells is largely lost in cells transfected with the ING2 RNAi vector. E) Cells transfected and incubated in growth or differentiation medium as described in Figure 2C were fixed and subjected to Hoechst staining to detect nuclei and indirect immunofluorescence to visualize the myogenic marker myosin heavy chain (MHC). Data show that ING2 knockdown leads to an overall reduction in myotube numbers in agreement with the results depicted in Figures 2C and 2D, and Figure S1D.
(TIF)

Figure S2 **Structure-function analysis of ING2’s effect on muscle differentiation.** A) ING2 indirect immunofluorescence of C2C12 cells cotransfected with a GFP expression construct together with an empty expression vector (−) or one encoding ING2 WT, ING2 ΔLZ, ΔC, or ΔPHD deletion mutant, or ING2 Y215A point mutant. Fixed cells were also stained with the Hoechst DNA dye. Micrographs depicting expressed ING2 (red), GFP (green), and nuclei (blue) in cells as visualized by fluorescence microscopy. ING2 was visualized by indirect immunofluorescence using the anti-ING2 antibody (161861-AP). B) Hoechst and GFP fluorescence images of C2C12 cells transfected with plasmids to express the myogenin-p-RFP myogenic reporter gene and GFP, as a marker of transfected cells, together with an empty expression vector (−) or one encoding wild type (WT) ING2 or one of three deletion mutants of ING2, as described in Figure 3D and grown under growth conditions. The fields shown in this figure are the same as those displayed in the upper panels of Figure 3D depicting RFP signals. GFP expression suggests equivalent transfection efficiencies between the different transfections. C and D). Analysis of myogenin-p-RFP intensity and number of nuclei in C2C12 myotubes at day 2 and day 3 of differentiation. C2C12 cells transfected with the myogenin-p-RFP reporter gene construct and GFP encoding plasmid together with an empty expression vector control (−) or one encoding wild type ING2 (WT), or ΔLZ, ΔC or ΔPHD deletion mutant ING2, were incubated in growth (day 0) or differentiation media for 2 or 3 days, and subjected to Hoechst staining followed by fluorescence microscopy to visualize myogenin-p-RFP, GFP and nuclei (see Figure 3D). Myotubes at day 2 and day 3 of differentiation were detected and analyzed by the Cellomics Kinetic Scan Reader and its associated myotube BioApplication based on myogenin-p-RFP fluorescence and Hoechst staining as a marker of nuclei. Within each identified myotube, nuclei number and the intensity of myogenin-p-RFP signal per myotube were determined. Total myotube numbers were obtained and multiplied by nuclei numbers or RFP-intensity per myotube to arrive at cumulative numbers of nuclei and RFP intensity in myotubes. 10 to 15 fields within each well of a 24-well plate were scanned. For each day 2 and day 3 of differentiation, values of the two parameters were normalized to a global average of data for each experiment and expressed relative to that of the control cells. Each column in the bar graphs represents the mean (± SEM) of relative value of intensity of fluorescence of myogenin promoter driven red fluorescence protein (C) or myotubes’ associated nuclei (D) at day 2 (clear rectangle) or day 3 (grey rectangle) of differentiation from five independent experiments. * indicates significant difference (p<0.05, ANOVA) against respective control. # indicates significant difference as compared to control (p<0.05, unpaired t-test).
(TIF)

Figure S3 ING2 association with Sin3A and HDAC1. A and B) ING2’s leucine zipper motif is important for ING2 association with HDAC1 and Sin3A. Comparison of association of wild type ING2 (WT) versus leucine zipper motif deleted ING2 (ΔLZ) with each of HDAC1 (A) and Sin3A (B) using coimmunoprecipitation assays on lysates of 293T cells cotransfected with ING2 and HDAC1 or Sin3A as described in Figures 5A and 5B. Quantified HDAC1 or Sin3A interacting with ING2 was normalized to the amount of immunoprecipitated ING2 (see Figures 5A and 5B). Normalized ING2-associating HDAC1 or Sin3A was then expressed relative to that obtained with the wild type ING2 control. Each column in the bar graph represents the mean (±SEM) relative HDAC1 (A) or Sin3A (B) interacting with ING2, of four independent experiments. Deletion of the leucine zipper significantly decreased ING2 association with HDAC1 and Sin3A (p<0.05, two-tailed, unpaired t-test). C) ING2 forms as a complex with HDAC1 and Sin3A in C2C12 cells. Lysates of C2C12 cells transfected with FLAG/tagged ING2, were subjected to sequential anti-FLAG immunoprecipitation, FLAG peptide elution, and immunoprecipitation of FLAG- immunocomplexes in eluates with mouse anti-Sin3A antibody (α-Sin3A IP, +) or mouse anti-IgG antibody (α-Sin3A IP, −), followed by ING2 (α-ING2), HDAC1 (α-HDAC1) and Sin3A (α-Sin3A) immunoblottings. D) Expression of ING2, HDAC1, and Sin3A in lysates used as described in C were confirmed by immunoblotting of the lysates with ING2, HDAC1, and Sin3A antibodies (Lane 1). The supernatant of the FLAG immunoprecipitation was immunoblotted for ING2 to determine degree of depletion of FLAG/ING2 (Lane 2). Untransfected lysates were also immunoblotted as a control (Lane 3). Anti-ING2 (11560-AP) antibody was used in the detection of ING2 in C and D. Tubulin immunoblotting was used as a loading control. Scans shown in C and D are from a representative experiment that was repeated two times.
(TIF)
