# Supplementary figures and images for: Identification of a Novel Function for the Chromatin Remodeling Protein ING2 in Muscle Differentiation
Source: PLoS One. 2012 Jul 12;7(7):e40684. doi: 10.1371/journal.pone.0040684 (PMC3395697; doi:10.1371/journal.pone.0040684)

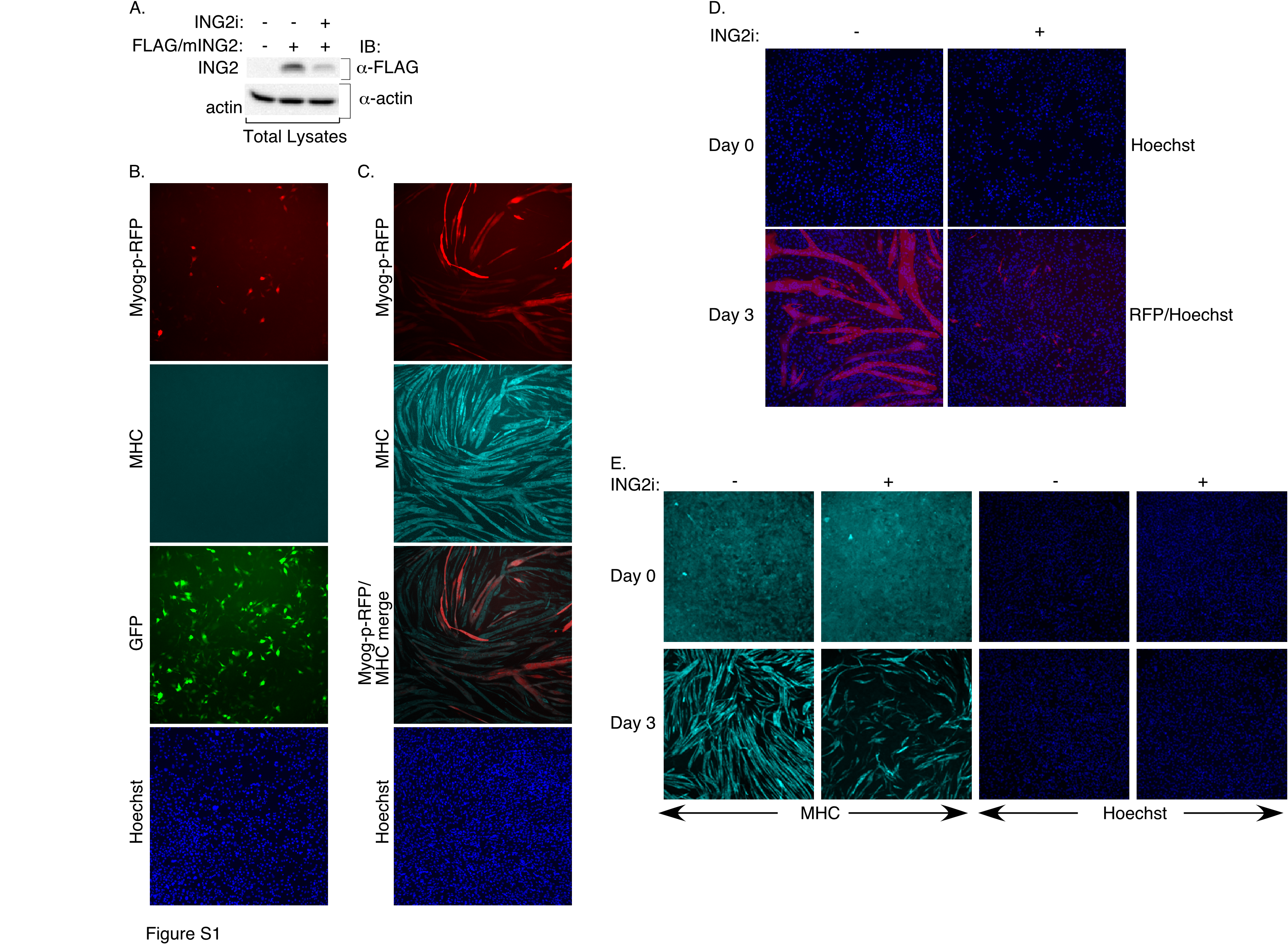

Supplement: Figure S1 — ING2 knockdown negatively regulates myogenesis. A) ING2 RNAi plasmid induces ING2 knockdown. Lysates of 293T cells transfected with empty vector (FLAG/mING2, −) or mouse ING2 expressing vector (FLAG/mING2, +) together with the RNAi vector control (ING2i, −) or ING2 RNAi (ING2i, +) plasmid, were subjected to FLAG and actin immunoblottings, with the latter serving as loading control. ING2 RNAi induces efficient reduction in ING2 protein levels. B and C) The myogenin-p-RFP reporter as a marker of myogenic differentiation in transfected cells. C2C12 cells transfected with a vector containing cDNA encoding the tdtomato-red fluorescent protein (RFP) under the control of the myogenin promoter together with the pEGFP(N1) plasmid to express green fluorescent protein (GFP) under the control of CMV promoter, were left in growth medium for another two days, and fixed then (B), or three days after switching to myogenic differentiation medium (C). Cells were labeled for the terminal differentiation marker myosin heavy chain (MHC) using indirect immunofluorescence and for nuclei using Hoechst DNA stain, and visualized by fluorescence microscopy (Material and Methods). B) Under growth condition only a small fraction of transfected cells (GFP) express RFP due to low myogenic differentiation as indicated by low myosin heavy chain signal. C) Upon differentiation, RFP expression is enhanced drastically and appears to be enriched in multinucleated myotubes as reflected by overlap with a subpopulation of the myosin heavy chain labelled myotubes. D) Scans relating to RFP and GFP fluorescence images of cells transfected with control and ING2 RNAi shown in Figure 2C. The scans in upper panel show cell numbers under growth conditions (Day 0) as indicated by Hoechst staining. The lower two panels are merged images of myogenin-p-RFP (same images as in lower panels of Figure 2C) and nuclei fluorescent signals of cells transfected with vector control RNAi (left panel) or with ING2 RNAi plasmid ( [file pone.0040684.s001.tif]

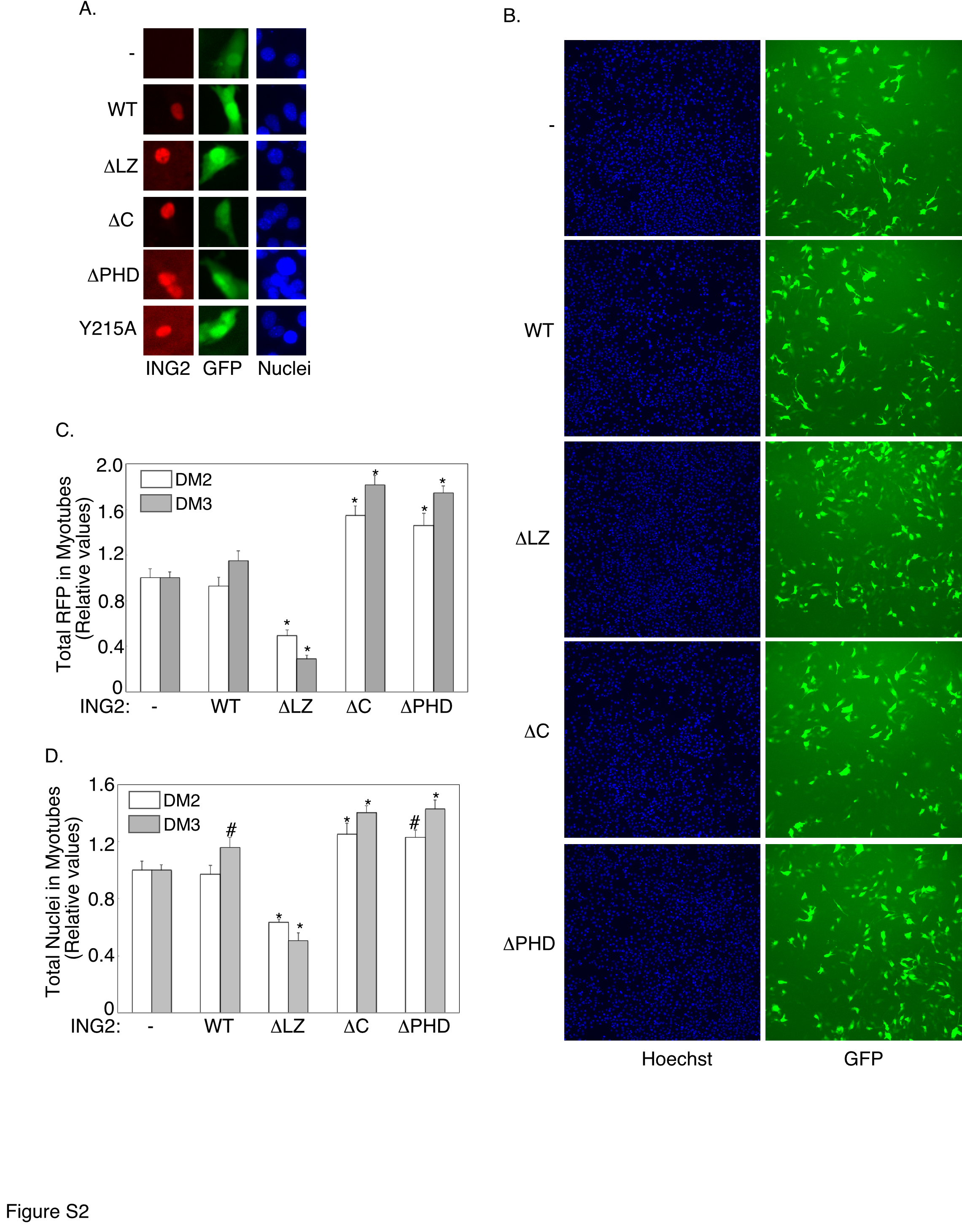

Supplement: Figure S2 — Structure-function analysis of ING2’s effect on muscle differentiation. A) ING2 indirect immunofluorescence of C2C12 cells cotransfected with a GFP expression construct together with an empty expression vector (−) or one encoding ING2 WT, ING2 ΔLZ, ΔC, or ΔPHD deletion mutant, or ING2 Y215A point mutant. Fixed cells were also stained with the Hoechst DNA dye. Micrographs depicting expressed ING2 (red), GFP (green), and nuclei (blue) in cells as visualized by fluorescence microscopy. ING2 was visualized by indirect immunofluorescence using the anti-ING2 antibody (161861-AP). B) Hoechst and GFP fluorescence images of C2C12 cells transfected with plasmids to express the myogenin-p-RFP myogenic reporter gene and GFP, as a marker of transfected cells, together with an empty expression vector (−) or one encoding wild type (WT) ING2 or one of three deletion mutants of ING2, as described in Figure 3D and grown under growth conditions. The fields shown in this figure are the same as those displayed in the upper panels of Figure 3D depicting RFP signals. GFP expression suggests equivalent transfection efficiencies between the different transfections. C and D). Analysis of myogenin-p-RFP intensity and number of nuclei in C2C12 myotubes at day 2 and day 3 of differentiation. C2C12 cells transfected with the myogenin-p-RFP reporter gene construct and GFP encoding plasmid together with an empty expression vector control (−) or one encoding wild type ING2 (WT), or ΔLZ, ΔC or ΔPHD deletion mutant ING2, were incubated in growth (day 0) or differentiation media for 2 or 3 days, and subjected to Hoechst staining followed by fluorescence microscopy to visualize myogenin-p-RFP, GFP and nuclei (see Figure 3D). Myotubes at day 2 and day 3 of differentiation were detected and analyzed by the Cellomics Kinetic Scan Reader and its associated myotube BioApplication based on myogenin-p-RFP fluorescence and Hoechst staining as a marker of nuclei. Within each identified myotube, nuclei number an [file pone.0040684.s002.tif]

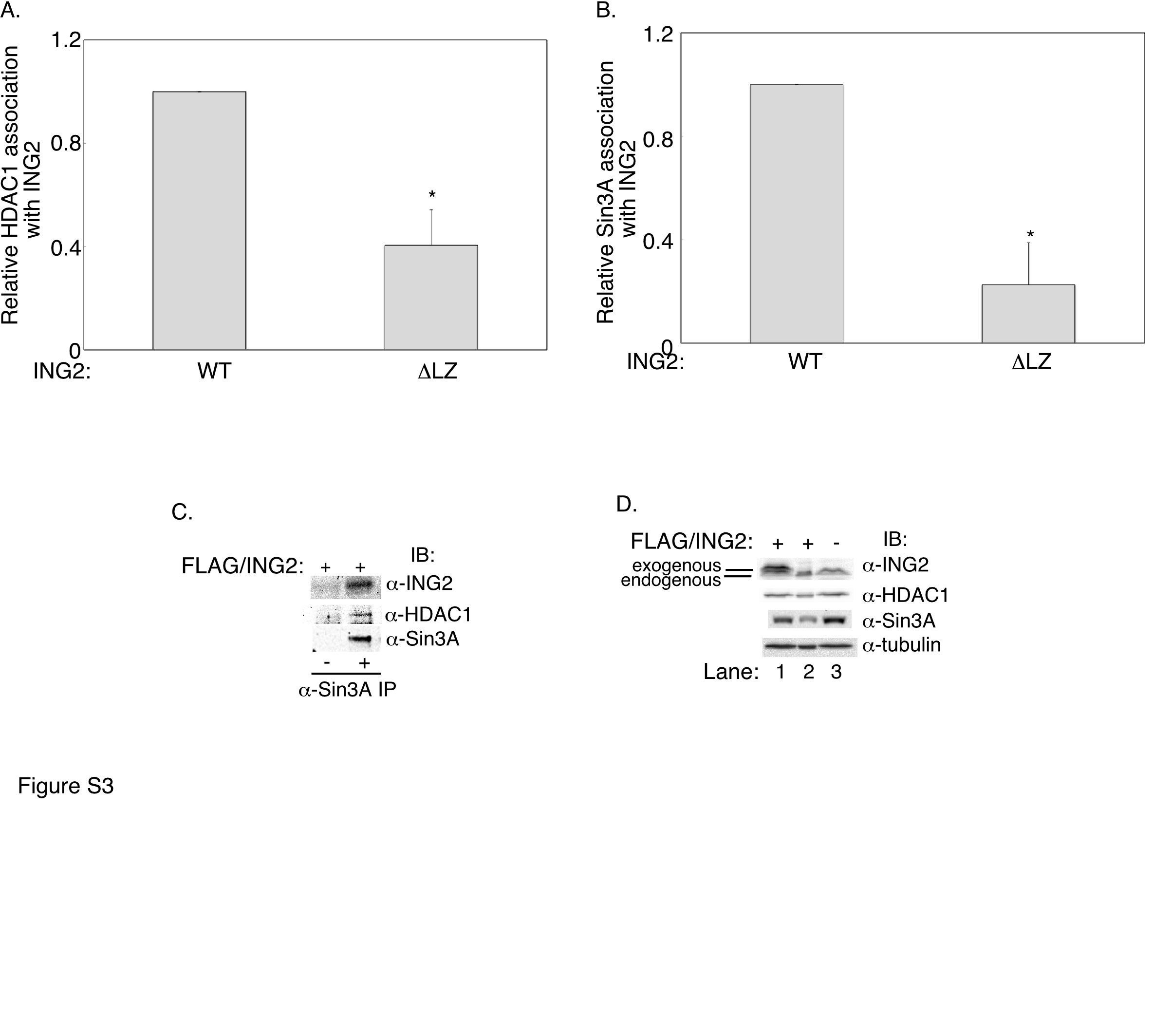

Supplement: Figure S3 — ING2 association with Sin3A and HDAC1. A and B) ING2’s leucine zipper motif is important for ING2 association with HDAC1 and Sin3A. Comparison of association of wild type ING2 (WT) versus leucine zipper motif deleted ING2 (ΔLZ) with each of HDAC1 (A) and Sin3A (B) using coimmunoprecipitation assays on lysates of 293T cells cotransfected with ING2 and HDAC1 or Sin3A as described in Figures 5A and 5B. Quantified HDAC1 or Sin3A interacting with ING2 was normalized to the amount of immunoprecipitated ING2 (see Figures 5A and 5B). Normalized ING2-associating HDAC1 or Sin3A was then expressed relative to that obtained with the wild type ING2 control. Each column in the bar graph represents the mean (±SEM) relative HDAC1 (A) or Sin3A (B) interacting with ING2, of four independent experiments. Deletion of the leucine zipper significantly decreased ING2 association with HDAC1 and Sin3A (p<0.05, two-tailed, unpaired t-test). C) ING2 forms as a complex with HDAC1 and Sin3A in C2C12 cells. Lysates of C2C12 cells transfected with FLAG/tagged ING2, were subjected to sequential anti-FLAG immunoprecipitation, FLAG peptide elution, and immunoprecipitation of FLAG- immunocomplexes in eluates with mouse anti-Sin3A antibody (α-Sin3A IP, +) or mouse anti-IgG antibody (α-Sin3A IP, −), followed by ING2 (α-ING2), HDAC1 (α-HDAC1) and Sin3A (α-Sin3A) immunoblottings. D) Expression of ING2, HDAC1, and Sin3A in lysates used as described in C were confirmed by immunoblotting of the lysates with ING2, HDAC1, and Sin3A antibodies (Lane 1). The supernatant of the FLAG immunoprecipitation was immunoblotted for ING2 to determine degree of depletion of FLAG/ING2 (Lane 2). Untransfected lysates were also immunoblotted as a control (Lane 3). Anti-ING2 (11560-AP) antibody was used in the detection of ING2 in C and D. Tubulin immunoblotting was used as a loading control. Scans shown in C and D are from a representative experiment that was repeated two times. (TIF) [file pone.0040684.s003.tif]
